# Biological Risk Factors Influencing Vascular Cognitive Impairments: A Review of the Evidence

**DOI:** 10.3390/brainsci13071094

**Published:** 2023-07-19

**Authors:** Silvina Iluț, Ştefan Cristian Vesa, Vitalie Văcăraș, Lavinia Brăiță, Vlad-Constantin Dăscălescu, Ioana Fantu, Dafin-Fior Mureșanu

**Affiliations:** 1 Department of Neurosciences, “Iuliu Haţieganu” University of Medicine and Pharmacy, 8 Victor Babeş Street, 400012 Cluj-Napoca, Romania; silvina.ilut@yahoo.com (S.I.); vvacaras@yahoo.com (V.V.); laviniabraita0810@gmail.com (L.B.); vlad.dascalescu2609@gmail.com (V.-C.D.); ioanafantu@gmail.com (I.F.); dafinm@ssnn.ro (D.-F.M.); 2 Clinical Rehabilitation Hospital, 46-50 Viilor Street, 400347 Cluj-Napoca, Romania; 3 Department of Pharmacology, Toxicology and Clinical Pharmacology, “Iuliu Haţieganu” University of Medicine and Pharmacy, 23 Gheorghe Marinescu Street, 400337 Cluj-Napoca, Romania; 4 RoNeuro Institute for Neurological Research and Diagnostic, 37 Mircea Eliade Street, 400364 Cluj-Napoca, Romania

**Keywords:** cognitive reserve, vascular risk factors, blood pressure variability, stroke

## Abstract

Vascular cognitive impairment encompasses several types of deficits, ranging from mild cognitive impairment to dementia. Cognitive reserve refers to the brain’s ability to balance damage and improve performance through certain types of brain networks. The purpose of this review was to assess the relationship between reserve in vascular impairment, specifically looking at whether cognitive impairment is influenced by cognitive reserve, identifying significant vascular risk factors and their pathological pathways. To achieve this purpose, a review covering these issues was conducted within the Embase, Cochrane, and PubMed database. A total of 657 scientific articles were found, and 33 papers were considered for the final analysis. We concluded that there is no consensus on the protective effects of brain reserve on cognitive impairment. Stroke and diabetes can be considered significant risk factors for vascular cognitive impairment, while hypertension is not as damaging as blood pressure variability, which structurally alters the brain through a variety of mechanisms.

## 1. Introduction

Cognitive impairment (CI) is a broad term that describes impairments in different cognitive domains, such as attention, memory, decision-making, planning, reasoning, knowledge, judgment, perception, language, understanding, and visuospatial function. One subtype of CI is vascular cognitive impairment that includes all kinds of cognitive deficits, from mild ones to dementia. On the one hand, mild cognitive impairment (MCI) refers to the early stages, in people who are still able to perform most activities of daily living independently. On the other hand, vascular dementia (VaD) is a type of dementia caused by brain damage due to decreased blood flow to the brain, in which people can lose, in certain stages, their independence. Vascular cognitive impairment is seen in patients with stroke, microinfarcts, microhemorrhages, loss of microstructural tissue integrity, strategic white matter tracts, and secondary neurodegeneration [1].

Cognitive reserve is a crucial concept that enables the brain to withstand insults and atrophies, thus delaying the emergence of symptomatic manifestations [2]. Following a vascular brain lesion, the intricate networks within the brain become compromised. There is an increasing recognition of the significance of targeting vascular risk factors in the prevention of dementia. Among the leading causes of dementia, Alzheimer’s disease (AD) is the most prevalent, followed by VaD [1].

Long-standing hypertension stands as a primary etiological factor for cerebrovascular diseases, contributing to both hemorrhagic and, more commonly, atherothrombotic strokes. In patients with chronic hypertension, the presence of atherosclerosis and multiple cerebral microinfarcts is highly characteristic. These lacunar infarctions result from the occlusion of tiny cerebral arteries. Furthermore, general arterial narrowing is frequently observed in individuals with chronic hypertension. Consequently, reduced collateral flow necessitates higher pressure to sustain proper blood flow in cerebral tissue. Thus, blood pressure variability, stemming from increased arterial stiffness or poor patient adherence to antihypertensive treatment, has been proposed as a significant cerebrovascular risk factor [3].

Cerebral small vessel disease is believed to play a pivotal role in the pathophysiology of MCI [4], further establishing the intricate relationship between blood pressure and dementia. Neurologists commonly refer to MCI as the presence of mild memory complaints that do not yet significantly impede daily functioning but are disproportionately concerning, given an individual’s education and age [5]. These complaints often serve as early indicators of dementia.

From a clinical perspective, the identification of treatable risk factors for vascular cognitive impairment holds paramount importance. However, the available evidence does not converge on a single identifiable factor [6]. Hypertension, diabetes, metabolic syndrome, stroke, and cardiac disease have all been implicated in progressive cognitive impairment, yet, a unified disease-modifying therapeutic strategy remains elusive [6].

Against this backdrop, the primary objective of this review paper is to explore the existing evidence pertaining to the relationship between cognitive reserve, risk factors, and cognitive impairment, with a specific focus on blood pressure variability and its underlying pathophysiological mechanisms.

## 2. Materials and Methods

In order to achieve the aforementioned goals, the subject was divided into two sections, each representing a different aspect: (1) cognitive reserve and ageing caused by vascular disease, (2) underlying pathophysiological link between blood pressure variability, stroke, diabetes, and cognitive impairment.

For these subjects, a systematic review was conducted in accordance with PRISMA-IPD (Preferred Reporting Items for a Systematic Review and Meta-analysis of Individual Participant Data) guidelines [6].

### 2.1. Search Strategies and Article Eligility

A search strategy for each section was devised which can be consulted in Table 1. Inclusion criteria was represented of observational studies, experimental studies, randomized control trials, and systematic reviews published in English after 2010, focusing on vascular cognitive impairment. Articles published as abstracts, focusing on genetic, infectious, or traumatic degenerative brain diseases and pediatric populations were not taken into consideration. With these criteria, a systematic article search was undertaken on the Embase, Cochrane, and PubMed databases. Additionally, references which were cited in included studies and considered relevant were taken into consideration.

### 2.2. Quality Analysis and Data Extraction

Each author searched databases using one of the search strategies mentioned above. After removing duplicate articles, the authors selected suitable articles in two stages. Firstly, the article was considered suitable for inclusion based on its title and abstract. Secondly, the full-text articles were read independently and considered into the selection if relevant to the subject. The article’s authors or the journal in which it was published did not have any influence whatsoever on the author’s decision to include it or not.

The data extracted from the selected articles, both quantitative and qualitative results, were narratively summarized by the authors.

## 3. Results

**Figure 1:**
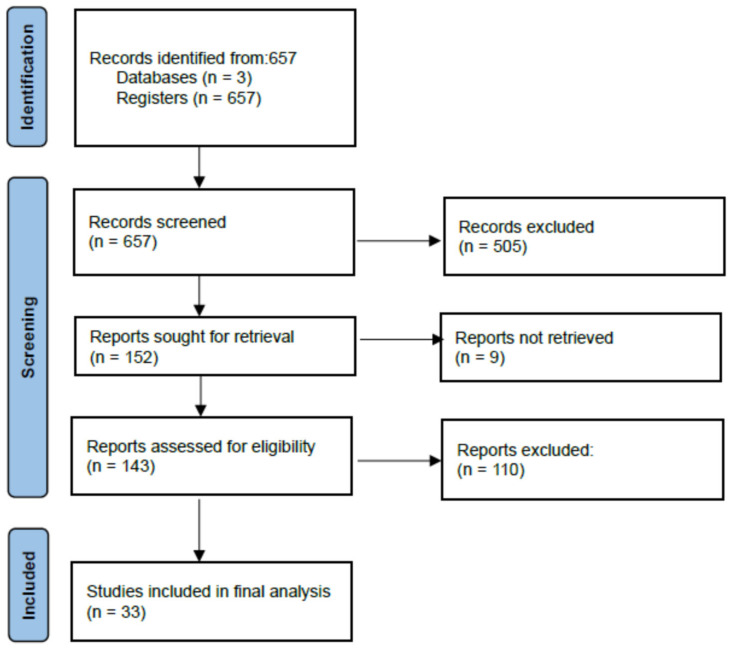
PRISMA diagram of the study’s selection process.

In total, after having applied the above search terms, a total of 657 scientific articles were found in the Embase, Cochrane, and PubMed search engines. After having removed duplicate articles and 547 papers after abstract and title screening, a total of 143 papers remained to be read in full. A total of 110 papers were read in full-text and excluded based on inclusion and exclusion criteria. A total of 33 papers were taken into consideration for the final analysis (Figure 1).

### 3.1. What Is the Relationship between Ageing Caused by Vascular Disease and Cognitive Reserve?

Some studies have shown that it is absolutely necessary that researchers evaluate the relationship between cognitive impairment and cognitive outcome, using the cognitive indicators to conclude the normal and pathological changes in brain function and structure [7]. Functional brain activity deals with the networks of the brain performing different tasks and the connections between them [8]. The flexibility and adaptability of the human brain allows the brain to cope better with age-related brain changes and with dementia [9,10].

Cognitive reserve is a continuously modifying factor that is related to the environment and life experiences [11,12], so, understanding the concept of the cognitive reserve could lead to a reduced speed of cognitive impairment with age and, at the same time, could diminish the risk of dementia. It has been observed that people with higher educational and occupational achievements have a reduced risk of developing Alzheimer’s disease [13,14,15]. Stimulating brain activities may reduce the risk of dementia by 40% [16].

Even if it is optimal to start healthy practices early in life, there is evidence that suggests that is not too late to start both physical and mental activity in old age [17].

The neuroplasticity substrates of the brain consist of neurogenesis, anti-oxidant defense, neurotrophic signaling, inflammation, and stress response. It is known that those substrates are modulated by diet and physical activity, while cognitive exercises strengthen cognitive reserve [18,19].

### 3.2. Is There an Underlying Pathophysiological Link between Blood Pressure Variability, Stroke, Diabetes, and Cognitive Impairment?

On the other hand, in two observational studies [20,21] on large populational groups, it was concluded that stroke and diabetes are the two risk factors for MCI, while arterial hypertension and cardiovascular disease where not found to have any statistical relationship to cognitive impairment. One observational study [22] concluded that antihypertensive treatment, when effective, has a beneficial effect on reducing cognitive impairment at 3 months after treatment initiation. Moreover, the largest observational study taken into consideration [23] suggests that type 1 diabetes with longer-lasting elevated HbA1c values and higher systolic blood pressure values has a negative effect on cognitive impairment. Other studies have demonstrated a positive association between blood pressure variability and cognitive impairment [24,25] regarding blood pressure and its involvement in neurocognitive functioning [26,27,28]. Although the exact pathological mechanism is currently under investigation, there is proof that blood pressure variability acts as an independent predictor for cognitive impairment and is responsible for using more than a single pathway [29]. Blood pressure fluctuations alter cerebral microvasculature through multiple mechanisms, damaging the structure of the cerebrovascular system [30,31,32]. Cerebral hypoperfusion [33], endothelial dysfunction, inflammation [34], oxidative stress and neurohormonal activation [35] are some of the known mechanisms involved in cognitive impairment correlated to blood pressure variability [36].

## 4. Discussion

The concept of cognitive reserve expresses the variability of the human being to be able to compensate for age-related brain changes or brain pathology [9]. Cognitive reserve encompasses the functionality, plasticity, and adaptability of the brain [7].

One of the age-related brain changes is CSVD, which is associated with the loss of white matter functionality, leading to disability and cognitive impairment [9]. CSVD is characterized by progressive white matter hyperintensities, which have been found to be associated with various risk factors [35]. Several studies [33] have focused on white matter intensities in CSVD, including 7893 patients, as well as 247 patients with cerebral autosomal dominant arteriopathy with subcortical infarcts and leukoencephalopathy. These studies revealed that higher cognitive reserve reduces the negative impact of white matter hyperintensities on cognition [9].

Dementia, a major neurocognitive disorder, encompasses numerous factors that cause cognitive impairment. It is believed to result from the overlapping of three processes [17]. The first process is age-related and referred to as “normal” cognitive impairment. The areas most affected by this decline, even since early adulthood, are those involving mental speed, volume of processing, and coordination efficiencies, such as attention, working memory, verbal recall, reasoning, multitasking, and response inhibition [37,38,39]. Conversely, vocabulary and knowledge of the world tend to be well preserved [40]. Additionally, there is a gradual loss of brain mass, with the hippocampus, caudate nucleus, putamen, and frontal cortex being the most affected regions over time [41,42]. As a consequence of these changes, the decline in white matter volume is most noticeable in the prefrontal region [43]. The second process involves pathological changes that worsen cognitive impairment. The last process refers to the brain’s ability to reallocate its resources.

The human brain possesses both structural and dynamic capacities to combat atrophies and lesions. There are believed to be two types of cognitive reserve. The first is a “passive” or static model, in which the network of neurons activates near the local damage. This mechanism is only utilized when there are sufficient functional and structural neurons. The second type is an “active” or dynamic model of cognitive reserve, in which the brain creates new neural circuits and recruits other regions to compensate for the reduced number of neurons [17]. Individuals with higher cognitive reserve tend to experience a more rapid decline when symptoms arise [17].

The main risk factors for vascular dementia (VaD) include age, hypertension, absence of antihypertensive medication, diabetes, cigarette smoking, history of cardiovascular disease (coronary heart disease, congestive heart failure, peripheral vascular disease), atrial fibrillation, left ventricular hypertrophy, hyperhomocysteinemia, orthostatic hypotension, cardiac arrhythmias, hyperfibrinogenemia, and sleep apnea [44,45].

Given the absence of modifying treatments for dementia, recent scientific research has focused on early cognitive impairment, particularly MCI [5]. While cardiovascular diseases and cognitive impairment (both MCI and dementia) are often comorbidities, sharing several risk factors such as smoking, obesity, and physical inactivity, it remains unclear whether cardiovascular diseases, vascular diseases, or events alone are attributable risk factors for the development of cognitive impairment. Disturbances in cerebral blood flow play a crucial role in the pathophysiological pathways leading to dementia, including Alzheimer’s dementia, hypertension, coronary artery disease (CHD), stroke (including cerebral infarction and cerebral hemorrhage), and metabolic syndrome with its components, including diabetes and obesity, which may be considered potential risk factors for MCI, among other factors [20,46].

Firstly, arterial stiffness and endothelial dysfunction, which are common in cardiovascular diseases, contribute to reduced cerebral blood flow and impaired vascular function in the brain, leading to cognitive impairment [47]. Hypertension, a major risk factor for cardiovascular diseases, has been associated with an increased risk of MCI and dementia [48]. The mechanisms underlying this association involve chronic cerebral hypoperfusion, oxidative stress, inflammation, and impaired clearance of amyloid-beta, a hallmark protein implicated in Alzheimer’s disease [49,50].

Secondly, cerebrovascular diseases, such as stroke and cerebral infarction, can lead to cognitive impairment and dementia. Ischemic stroke can cause focal brain damage, disrupting cognitive functions depending on the affected brain regions [51]. Additionally, silent cerebral infarcts, which are often asymptomatic, have been associated with an increased risk of cognitive impairment and dementia [52].

Furthermore, chronic systemic inflammation and oxidative stress, characteristic of cardiovascular diseases, can contribute to neuroinflammation and neurodegeneration, thereby increasing the risk of cognitive impairment [53,54]. Inflammatory markers, such as C-reactive protein (CRP) and interleukin-6 (IL-6), have been found to be elevated in individuals with MCI and dementia [55,56].

In summary, there is growing evidence suggesting that cardiovascular diseases and risk factors are associated with an increased risk of cognitive impairment, including MCI and dementia. The mechanisms linking these conditions involve cerebral hypoperfusion, vascular dysfunction, chronic inflammation, oxidative stress, and neurodegeneration. Identifying and managing cardiovascular risk factors early on may help reduce the risk of cognitive impairment and promote brain health.

## 5. Conclusions

To conclude, the current literature presents contradictory data regarding the protective effects of brain reserve on cognitive impairment in the presence of vascular changes. Further research is needed to fully understand the role of cognitive reserve in mitigating cognitive impairment. While the precise risk factors for vascular cognitive impairment remain uncertain, studies suggest that stroke and uncontrolled diabetes are modifiable risk factors, whereas hypertension appears to have less influence compared to oscillatory blood pressure values. Blood pressure variability has detrimental effects on the cerebral microvasculature, causing structural damage at a molecular and cellular level, thereby impairing cognitive function and overall health. In addition to managing modifiable risk factors and promoting healthier lifestyle choices, the current literature emphasizes the importance of continuous physical and cognitive training as beneficial for individuals with mild cognitive impairment.

## Figures and Tables

**Table 1:** Research questions and their strategies.

Research Questions (RQs)	Search Strategy
RQ 1: What is the relationship between ageing caused by vascular disease and cognitive reserve?	cognitive reserve AND cognitive ageing
RQ 2: Is there an underlying pathophysiological link between blood pressure variability, stroke, diabetes, and cognitive impairment?	cognitive reserve AND cognitive ageing and adults and blood pressure cognitive reserve AND cognitive ageing and adults and stroke cognitive reserve AND cognitive ageing and adults and diabetes

## Data Availability

Not applicable.
